# Strangulated Hernia—V

**Published:** 1908-11-21

**Authors:** 


### Surgery.

### STRANGULATED HERNIA—V.

This condition must be classed among surgical emergencies; that is to say, the life of the patient is endangered by it unless relief is immediately obtained by surgical means. A hernia may be strangulated when it first makes its appearance, but strangulation is more often observed in one which has existed for some time and of whose presence the patient is perfectly well aware. It is on account of this that the presence of a hernia cannot be regarded lightly, so that it may spoil what is an otherwise first-class life for insurance purposes. There can be no doubt that cases of strangulated hernia are less rare than they used to be. This is probably due to the fact that a radical cure is nowadays performed in nearly all cases of simple hernia, unless there is some definite contra-indication, whereas previously reliance was placed upon truss pressure as a means of control; but sooner or later the truss became weak or inefficient and the conditions were then favourable for strangulation to ensue.

Many cases of simple irreducible or obstructed herniæ are diagnosed as being strangulated; but to constitute genuine strangulation three factors must be present. They are: (1) an impediment to the return of the extruded coils; (2) interference with their vascular supply; and (3) intestinal obstruction.

Strangulation may occur in one of many ways. For instance, it is easy to understand that if a hernia is already irreducible or obstructed, an extra coil of gut may be forced down into it by coughing or by exertion, which will produce uncomfortable pressure at the point of exit which is unyielding. Or, again, a hernia may be twisted in its descent so that the vessels in its mesentery are interfered with. But most cases can be explained on a more simple hypothesis. A coil of gut is forced down through a narrow unyielding aperture. Its blood-vessels are pressed upon and damaged. As a result gaseous interchange between the contained gases and the capillary vessels comes to a standstill; the extruded coils become blown up, the constriction at the orifice becomes greater still, and so a vicious circle is set up. It is not actually necessary therefore to suppose that the extruded coils are full when they descend or that the stagnation of their contents is the cause of the condition. The coils themselves may actually be empty; but there is, of course, intestinal obstruction at the point of constriction.

The symptoms caused are essentially the same whether the hernia be a femoral or an inguinal one; in the former case the constricting agent is the sharp edge of Gimbernat's ligament, while in the latter the neck of the sac itself is responsible. This is another argument, if one were needed, for regarding the sac of a hernia as a preformed anomaly. For it can only be supposed that the neck of the sac can have become thick and rigid enough to strangulate the hernia which it contains, if it has already been in position for some length of time, during which it has been subjected to chronic compression or irritation as it lies in the inguinal canal. If the sac was pushed down by the hernia and descended at the same time, we should expect the walls of the inguinal canal to be the constricting agent. But this is rarely, if ever, found to be the case. In some rare cases the whole hernia, sac and all, has been reduced by taxis but the symptoms have continued unrelieved, and the autopsy has revealed that the neck of the sac was still contricting the contained intestine.

The onset of symptoms is sudden, and is often associated with a violent effort as, for example, lifting a weight or coughing. But in rare cases it occurs when the patient is at rest or even asleep. The first thing which is noticed is acute pain in the hernia, which is irreducible, whether it was down before or now presents itself for the first time. The patient usually vomits and exhibits all the symptoms which we are accustomed to class together under the title of shock. As a general rule these symptoms are not so severe in a large hernia or in an old-standing one as they are if it be a small recent one; and, again, the symptoms may be much modified in an aged person, so much so that the diagnosis may be overlooked in such patients by the unwary.

Attention is usually drawn to the hernia by the patient himself, who complains of pain in it. A hard, tense, painful tender swelling will be found. No impulse at all can be obtained when the patient coughs. Contrast this with an obstructed hernia, in which a faint impulse can generally be obtained at the neck, or with the inflamed variety in which the impulse is not impaired. On percussing the hernia, if the patient can bear it, a dull note will be obtained. This is due to the presence of fluid in the sac; for this consists of damaged peritoneum and it reacts to the injury which it has received by secreting a serous effusion. In some cases the effusion may be hæmorrhagic in character, and the scrotum or skin covering the hernia will be discoloured accordingly.

The symptoms of acute intestinal obstruction supervene after a varying interval, generally a short one. Neither fæces nor flatus are passed and vomiting, of a nature different to that which is the result of the shock, sets in. First gastric, then biliary, and finally stercoraceous material is violently ejected with much straining. If the case is watched, the abdomen gradually becomes distended. As a general rule the higher in the alimentary tract the constriction is situated the sooner the vomiting begins. Conversely the early appearance of abdominal distension may be regarded as evidence that the strangulation is in the lower end of the small intestine or even in the colon itself.

When the symptoms here detailed present themselves the case is necessarily an urgent one, and no time should be lost in trying palliative measures, since operation is the only certain means of effecting a cure.

